# A 14-Day Therapeutic Exercise Telerehabilitation Protocol of Physiotherapy Is Effective in Non-Hospitalized Post-COVID-19 Conditions: A Randomized Controlled Trial

**DOI:** 10.3390/jcm12030776

**Published:** 2023-01-18

**Authors:** Cleofas Rodriguez-Blanco, Carlos Bernal-Utrera, Ernesto Anarte-Lazo, Juan Jose Gonzalez-Gerez, Manuel Saavedra-Hernandez

**Affiliations:** 1 Physiotherapy Department, Faculty of Nursing, Physiotherapy and Podiatry, University of Seville, 41009 Sevilla, Spain; 2 Fisiosur I+D Research Institute, 04630 Garrucha, Spain; 3 Doctoral Program in Health Sciences, University of Seville, 41009 Seville, Spain; 4 Department Nursing, Physiotherapy and Medicine, University of Almeria, 04120 Almeria, Spain

**Keywords:** exercise, COVID-19, long COVID, physiotherapy, quality of life

## Abstract

The emergence of COVID-19 has led to serious public health problems. Now that the acute phase of the pandemic has passed, new challenges have arisen in relation to this disease. The post-COVID-19 conditions are a priority for intervention, as months after the onset of the disease, they continue to present symptoms, especially physical and respiratory symptoms. Our aim is to test the efficacy of a fourteen-day telerehabilitation program of respiratory and strength exercises in people with post-COVID-19 conditions. For this purpose, a randomized controlled trial was generated in which data from 48 patients were analyzed using the BS, 30STSTST, MD12, VAFS, and 6MWT tests. The obtained results showed the benefit of the intervention in generating great results with respect to the control group.

## 1. Introduction

The COVID-19 pandemic has generated changes of unimaginable magnitude, and the economic, psychological, and scientific impact on our societies has been destabilizing [1], leading to millions of infections, home confinements, hospital and intensive care units (ICU) admissions, and deaths [2]. One of the major concerns derived from this pandemic is the high prevalence of post-COVID-19 symptomatology. Although the definition is evolving, it has been accepted that post-acute COVID-19 includes the persistence of symptoms or development of sequelae beyond 3 or 4 weeks from the onset of acute symptoms of COVID-19 [3].

This condition has usually been described to happen in COVID-19 patients admitted to the ICU; nonetheless, it has been claimed that not only hospitalized patients but also non-hospitalized and cared for at-home patients also suffer from this symptomatology [4,5], including reduced pulmonary and physical function, leading to a reduction in quality of life and emotional distress. In that sense, persistent symptoms such as fatigue, joint pain, dyspnea, and chest pain, among others, have been reported [5]. Nonetheless, although COVID-19 manifestations are well-documented, we still do not have the possibility to predict the potential long-term implications.

Exercise has been claimed to be essential in the management of post-COVID-19 sequelae [6] and guidelines for physical therapists and rehabilitation services have been published since the beginning of the pandemic [7]. Since several pathophysiological mechanisms have been described in post-COVID-19 conditions [8,9], several mechanisms have been suggested in relation to the possible mechanisms that could explain the benefits of implementing physical exercise in these patients, such as boosting the immune function, improving physical function, or improving respiratory capacity [9,10,11]. Therefore, rehabilitation seems to be a key point in the management of post-COVID-19 conditions [12]. 

Recently, it was proven that different exercise interventions performed through telerehabilitation methods, based on strength and breathing exercises, were effective in improving health status in confined patients with COVID-19, although breathing exercises were more effective for improving respiratory function [13,14]. In addition, this kind of intervention was well-received and recognized as a valuable tool by patients [15]. However, no other randomized controlled trials have been performed investigating the role of a telerehabilitation program based on both types of exercises in non-hospitalized patients with post-COVID-19 conditions.

The research question for this study was:

- Does a therapeutic exercise telerehabilitation protocol based on strength and respiratory exercises produce benefits for people with post-COVID-19 conditions?

## 2. Materials and Methods

### 2.1. Design

A randomized, controlled, parallel, double-blinded, two-arm clinical trial was conducted in Spain from April to June 2021. The trial is registered in the Australian–New Zealand Clinical Trials Registry with the number ACTRN12621000347864. The study was approved by the ethics committee of University Hospitals Virgen Macarena and Virgen del Rocio and complied with the Helsinki Declaration.

### 2.2. Participants and setting

We carried out a patient recruitment campaign, through public information in the media (radio, television, and internet), requesting the participation of persons with post-COVID-19 conditions, who had been diagnosed by their doctor and had a positive PCR test more than 40 days ago. All patients who expressed interest were subsequently informed in greater detail. The initial screening was carried out by telephone, after a recruitment period of three months. Before enrolling in the study, the patients gave their consent on the website (www.fisiosurid.com/covid19/: accessed on 1 April 2021) to comply with ethical and legal privacy concerns.

The participants were considered eligible between 18 and 75 years of age and were cases diagnosed positive for COVID-19 by PCR (polymerase chain reaction) test or antigen test by the epidemiology services, and additionally manifested symptoms attributed to COVID-19 by medical services for at least 40 days [3].

The exclusion criteria were evaluated by a physician through a video call interview and were the following: participants who required hospital admission for COVID-19, cardiovascular or hypertension without medical treatment, chronic lung or kidney diseases, chronic neurological or mental disorders, grade III osteoporosis, and acute phase disorders (rheumatological and vertebral disc abnormalities). Patients who had suffered from respiratory or musculoskeletal disease in the last 12 months (other than COVID-19) and were not fully recovered, and those with signs of serious illness or red flags (night pain, severe muscle spasm, unintentional weight loss, imbalance of symptoms) without control by a health professional were also excluded.

We randomized the participants by simple randomization, through free online software (http://www.randomized.org/: accessed on 14 April 2021). We obtained the randomization sequence before baseline testing began. The principal investigator and an external auditor obtained and guarded the randomization sequence. Participants and evaluators were unaware of the existence of this randomization sequence, so they never had access to it. We hid and guarded the randomization sequence to guarantee correct randomization with security.

Additionally, the evaluators were unaware of the random distribution of the participants, so they were blinded throughout the process. Participants and therapists were not blinded to treatment due to the nature of the interventions. Still, assessors and participants were blinded to group assignment, so the study design was double-blind.

### 2.3. Outcome Measures

All data were recorded using digital communication applications (WhatsApp or by email) with the patients on the first (day 1) and last day (day 14) of the intervention and were collected by researchers instructed in all protocolized procedures. The evaluators made appointments with the patients, and the evaluations were carried out via videoconference, with the following outcome measures:

A. Visual Analog Fatigue Scale (VAFS). This is a self-report scale measured from 0 to 10, and it is a valid and reliable instrument for the quantitative assessment of fatigue [16], where a higher score indicates a worse score, although there are no minimal clinically significant differences for patients with respiratory pathologies for VASF. Participants could download the form at the following address: https://www.fisiosurid.com/wp-content/uploads/2020/11/ESCALA-VISUAL-ANALOGICA-FATIGA.pdf: accessed on 1 April 2021. The evaluators, via video call, asked the patients what the VASF value they considered adequate was, on day 1 and day 14.
B. Six-Minute Walk Test (6MWT). It consists of recording the number of steps through the “StepsApp”, using the patient’s smartphone, and performing the following procedure: The evaluators asked the participants to walk as far as possible at home without generating 180° changes of direction, minimizing variability in the distribution of dwellings. The evaluators received the data recorded by the participants after performing the test, which can correctly determine the functional status [17]. The minimal clinically significant difference represents 54 m, or 75 steps [18]. A higher score indicates a better result on the test.
C. Thirty-Second Sit-to-Stand Test (30STST). The following procedure was carried out to standardize the test: The evaluators asked the participants to place a chair without arms with a straight back and a hard seat, stabilizing it against a wall (height from the floor to the seat would be between 45 and 50 cm). Seated participants were asked to keep their feet flat on the floor and keep their arms across their chest without moving them during all trials. They would then stand up fully and sit down once without using their arms. Participants will start the test sitting in a chair and, when instructed through the online application, will get up and then sit back down as many times as possible in a 30 s period. This test is a valid tool and is reliable for evaluating the performance of the peripheral muscles of the lower limbs [19]. The participants performed the test, and the evaluator counted the number of repetitions and the minimum clinically significant difference [20]. A higher score indicates a better result on the test.
D. Multidimensional Dyspnea-12 (MD12). We have applied this test, in its validated Spanish version, since it is a valid and reliable instrument to study the multidimensional character of dyspnea [21], with a minimum clinically significant difference of 2.83 points [22]. A higher score indicates a worse result on the test. Participants could download the form at the following address: https://www.fisiosurid.com/wp-content/uploads/2023/01/CUESTIONARIO-DISNEA-12.pdf: accessed on 1 April 2021. The evaluators, via video call, asked the participants to answer the questions on the scale, on day 1 and day 14.
E. The modified Borg Scale of perceived effort (BS) [23]. This scale provides the criteria to adjust to the intensity of the exercise, that is, to the workload, and thus anticipate and dictate the different powers of activity in sports and medical rehabilitation. It measures the entire range of activities that the individual perceives when performing exercise, with a minimum clinically significant difference of 0.9 points [24]. A higher score indicates a worse result on the test. Participants could download the form at the following address: https://www.fisiosurid.com/wp-content/uploads/2020/11/ESCALA-BORG.pdf: accessed on 1 April 2021. The evaluators, via video call, asked the patients what the Borg Scale value they considered adequate was, on day 1 and day 14.

The evaluation protocol was as follows:

1. The VAFS was assessed to determine the patient’s level of fatigue.
2. The participants performed the 6MWT test and the 30STST test.
3. The dyspnea and effort perceived were assessed by means of the MD12 and the BS, respectively.

All participants’ information was collected and classified by the evaluators, transferring the data to encrypted file sheets using Excel software (Excel, Microsoft, Redmond, WA, USA). The encryption of the Excel files was only known to the evaluators and the principal investigator, who had exclusive access to them. This information was updated through a secure encrypted online cloud, located on a secure encrypted online network. Additionally, we recorded the rate of loss to follow-up and its related causes. 

### 2.4. Interventions

Participants followed their assigned directions for 14 days, depending on the group to which they were assigned. Participants in the experimental group (EG) performed a 14-day therapeutic exercise program and patients in the control group (CG) performed relative home rest, consisting of their daily activities for daily living, without associated physical efforts. All study participants (EG and CG) were instructed that they could not perform any other physiotherapy treatment or sports physical activity simultaneously since any interference in the treatment would lead to exclusion from the study.

Group 1: Experimental group (EG). Physical therapy via 14-day therapeutic exercise telerehabilitation protocol

The 14-day therapeutic exercise telerehabilitation protocol consisted of 10 breathing- and strength-based exercises. This resistance and strength program aims to increase physical deconditioning and physiological deterioration in participants with post-COVID-19 conditions.

The evaluators informed the participants of the website where the exercises were hosted, available at: https://www.fisiosurid.com/ejercicios-tonificacion-respiratoria/: accessed on 1 April 2021. The participants performed the exercises at home once a day, for 14 days, with 12 repetitions per exercise each day for 30 min, although the repetition number could be modified after the intra-intervention evaluation, adapting according to the results of the BS scale. The exercise program was controlled and reinforced by a physical therapist through telematic control via videoconference with each participant.

Group 2: Control group (CG). Relative rest at home

The participants in the control group were only assessed, no intervention was performed, and they only had relative home rest, consisting of their daily activities of daily living, without associated physical efforts. These assessments were performed by a physiotherapist who was unaware of the group to which the participant belonged. Once the control group participants finished their participation in our study, we recommended that they perform the same exercise program as the experimental group, to improve their health status and avoid the ethical aspects associated with the control group.

### 2.5. Sample Size Calculation

Accepting an alpha risk of 0.05 and a beta risk of 0.1 in a two-sided contrast, 21 subjects were required in each group to detect a minimum difference of 16% (0.16) between the two groups, which is based on a previous pilot study [25], assuming that there are 2 groups and a standard deviation of 15% (0.15). A loss rate of 10% (0.1) has been estimated. Finally, at least 42 subjects were required to be included in the study. Software Granmo online (version 7.12) was used for sample size calculation.

### 2.6. Data Analysis

Statistical analysis was carried out using SPSS v. 26.0 (IBM, Armonk, NY, USA). We carried out the statistical analysis through a descriptive analysis of the data before the intervention, as a baseline. All the study variables were quantitative, except the gender variable, for which the normality was analyzed with the Chi-square test. The distribution of the rest of the quantitative variables was analyzed with the Kolmogorov–Smirnov normality test (Table 1). The within-group analysis was performed by applying the paired *t*-test (Table 1). The independent *t*-test was used to assess differences between groups (Table 1). The effect sizes were analyzed through the R-square coefficient (R^2^) (Table 1), considering effect sizes lower than 0.01 as small, higher than 0.06 as medium, and higher than 0.14 as large. The statistical analysis was conducted at a 95% confidence level. A *p*-value of less than 0.05 was considered statistically significant in all analyses. 

## 3. Results

**Figure 1:**
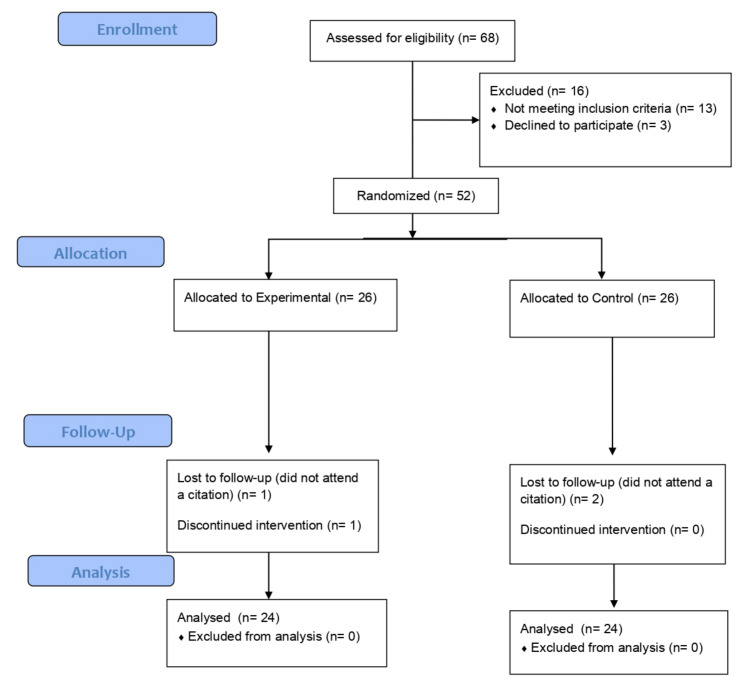
CONSORT flow diagram.

After conducting 68 evaluations, 52 participants met the inclusion criteria and were enrolled in the study, and 48 completed the trial (92%). There were two losses in the control group and two losses in the experimental group. The CONSORT flow diagram can be seen in Figure 1.

**Figure 2:**
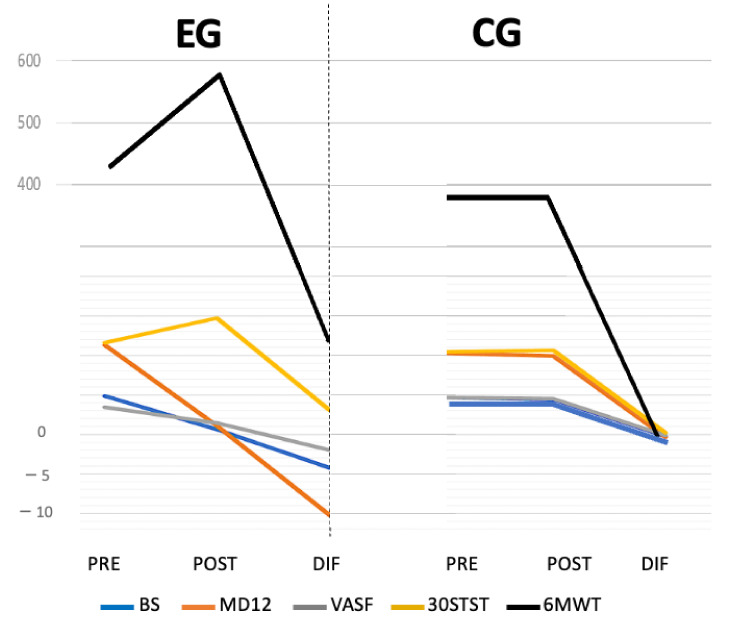
Comparative graph of pre–post-intervention values and intergroup differences. EG: experimental group; CG: control group; PRE: pre-intervention; POST: post-intervention; DIF: Pre–post-intervention differences data; BS: Borg Scale; MD12: Multidimensional Dyspnea-12; VAFS: Visual Analog Fatigue Scale; 6MWT: Six-Minute Walking Test; 30STST: 30-Second Sit-to-Stand Test.

In our study, there were no between-group differences in any outcome measures at baseline, so all groups were considered comparable. The Chi-square normality tests for the gender variable showed a normal distribution (*p* > 0.05), and the normality tests for the rest of the quantitative variables were of a normal distribution as well (*p* > 0.05). Our 14-day therapeutic exercise protocol was carried out, which induced statistically significant improvement between groups (Figure 2) and within groups in the experimental group (*n* = 24) with respect to the control group (*n* = 24), in all the study variables. Table 1 summarizes baseline and pre–post-intervention differences. Table 2 summarizes within-group and between-groups differences and effect size data for both groups.

We have obtained intragroup differences in the experimental group (*p* < 0.05) in all the variables studied, compared to the control group, which has not obtained significant intragroup differences (*p* > 0.05) in any study variable (Table 2), which means that our exercise protocol could induce objective changes in the BS, MD12, VAFS, 30STST, and 6MWT variables. No side effects were recorded during the implementation of the intervention.

Likewise, we have observed a possible relationship with the clinical effect of our protocol, analyzing the size of the effect, since we observed that all the values obtained were considered greater than large (R^2^ > 0.14), with the greatest effects being obtained for the variables BS (*p* < 0.001; F_1.75_ 103.906; R^2^ 0.693), MD12 (*p* < 0.001; F_1.75_ 52.044; R^2^ 0.531), and 30STST (*p* < 0.001; F_1.75_ 21.341; R^2^ 0.317) (Table 2).

## 4. Discussion

The present study demonstrated large differences in clinical outcomes between an exercise program based on strength and breathing exercises and the control group in participants with post-COVID-19 conditions. 

According to data achieved by the UK’s Office for National Statistics (ONS), from 20,000 subjects who tested positive, consisting of 90% non-hospitalized, 13.7% showed persisting symptomatology after 12 weeks of evolution [26], and this points to the importance of the treatment of these patients. Currently, only three studies in long-COVID-19 patients have been published, but none of them have a randomized controlled design [27,28,29]. These studies used the same telerehabilitation methods (videoconferencing) as well as similar therapeutic exercise protocols, based on breathing and toning exercises. As in our study, all presented favorable results in all cardiorespiratory measures and physical tests evaluated [27,28,29], and the effect sizes of the treatments vary depending on the study, being medium [27,29] and large [28].

In addition to high values achieved in our findings regarding the associated effect size, which could indicate critical clinical implications of the proposed intervention, previously it was recorded that patients involved in a similar telerehabilitation program reported positive impressions and considered that it was a great approach not only for being used during periods of home confinement, but also for being implemented for usual health interventions [14]. In that sense, telerehabilitation interventions evaluating the clinical effects in other samples such as subjects with respiratory or cardiac disorders have also found positive results [30,31]. 

Considering the multisystemic disturbances that may underlie post-COVID-19 symptoms, affecting immune, cardiac, respiratory, neurological, and musculoskeletal systems, among others, it is worth considering exercise as a great intervention to modulate symptomatology and to be included in the rehabilitation program of these patients, since it has already been demonstrated that it can act as a systemic polypill [32].

The potential strengths of the study are the randomized design, high treatment adherence, limited loss to follow-up, validated outcome measures, detailed process evaluation, and a methodology of assessment and intervention already performed and well-received by patients. The study had some limitations in that we only performed a short-term follow-up, when the exercise program finished. Therefore, according to our results, we cannot discuss possible benefits in the medium and long term in the subjects included in the intervention group, although we affirm that there are clinically significant differences when applying a protocol of 14 days of exercises, compared to rest or absence of physical activity as in our control group. Finally, we would like to point out that, although increasingly studied, remote assessments could become a limitation to extrapolate our findings to clinical practice and when evaluating telerehabilitation tools compared to face-to-face evaluations. Moreover, remote assessments depend on the subject’s praxis for self-assessment. Thus, some populations could have problems in managing these tools, such as elderly people. Future studies should assess the reliability of these kinds of interventions, comparing them with the same exercise programs performed in person. 

Finally, we would like to point out that although vaccines have improved the prognosis of patients with post-COVID-19 conditions, infections continue to happen, and people continue to develop symptomatology associated with COVID-19 [33]. Therefore, we think that our results are of interest since the intervention performed could be adopted in these subjects to improve their health status. 

## 5. Conclusions

A 14-day therapeutic exercise program performed through telerehabilitation devices based on strength and breathing exercises has been demonstrated to be effective in improving physical and respiratory function post-COVID-19. 

## Figures and Tables

**Table 1:** Baseline and pre–post-intervention differences. Data are expressed as mean (standard deviation) [95% CI Lower to Upper]. Gender is expressed as mean (percentage). BS: Borg Scale; MD12: Multidimensional Dyspnea-12; VAFS: Visual Analog Fatigue Scale; 6MWT: Six-Minute Walking Test; 30STST: 30-Second Sit-to-Stand Test; PRE: Pre-intervention data; POST: Post-intervention data; DIF_: Pre–post-intervention differences data. EG: experimental group; CG: control group.

	EG (*n* = 24)			CG (*n* = 24)		
	PRE	POST	DIF	PRE	POST	DIF
Age	38.75 (15.40) [32.24 to 45.26]			42.58 (11.40) [37.77 to 47.40]		
Gender	♂ 11 (22.91) ♀ 13 (27.08)			♂ 11 (22.91) ♀ 13 (27.08)		
Height	166.46 (7.97) [163.09 to 169.82]			165.83 (9.27) [161.91 to 169.74]		
Weight	73.53 (18.33) [65.79 to 81.27]			79.77 (20.99) [70.90 to 88.62]		
BMI	25.66 (1.78) [24.91 to 26.41]			25.74 (1.77) [24.99 to 26.49]		
BS	4.87 (2.11) [3.98 to 5.77]	0.62 (0.65) [0.35 to 0.90]	−4.25 (1.85) [−5.03 to −3.46]	4.67 (1.95) [3.84 to 5.49]	4.42 (1.84) [3.64 to 5.19]	–0.25 (0.53) [−0.47 to −0.02]
MD12	11.29 (7.54) [8.11 to 14.48]	1.08 (1.53) [0.44 to 1.73]	−10.21 (6.66) [−13.01 to −7.40]	10.29 (6.82) [7.41 to 13.17]	9.92 (6.59) [7.13 to 12.70]	−0.37 (0.65) [−0.64 to −0.10]
VAFS	3.42 (2.57) [2.33 to 4.50]	1.42 (1.84) [0.64 to 2.19]	−2.00 (2.28) [−2.96 to −1.03]	4.67 (2.26) [3.71 to 5.62]	4.50 (2.15) [3.59 to 5.41]	−0.17 (0.70) [−0.46 to 0.12]
6MWT	429.63 (192.50) [348.34 to 510.91]	577.54 (153.04) [512.92 to 642.16]	147.92 (165.57) [78.00 to 217.83]	379.46 (131.28) [324.02 to 434.89]	379.08 (131.37) [323.61 to 434.56]	−0.37 (10.67) [−4.88 to 4.13]
30STST	11.63 (2.39) [10.61 to 12.64]	14.71 (4.24) [12.92 to 16.50]	3.08 (2.80) [1.90 to 4.26]	10.42 (2.48) [9.37 to 11.47 ]	10.63 (2.70) [9.49 to 11.76]	0.21 (1.22) [−0.30 to 0.72]

**Table 2:** Within- and between-group differences and effect sizes. BS: Borg Scale; MD12: Multidimensional Dyspnea-12; VAFS: Visual Analog Fatigue Scale; 6MWT: Six-Minute Walking Test; 30STST: 30-Second Sit-to-Stand Test; DIF_: Pre–post-intervention differences data. EG: experimental group; CG: control group. Effect sizes are expressed as R-square coefficients (R^2^).

	DIF	DIF
	EG (*n* = 24)	CG (*n* = 24)
BS	*p* < 0.001	*p* > 0.05
	R^2^ 0.693	
MD12	*p* < 0.001	*p* > 0.05
	R^2^ 0.531	
VAFS	*p* < 0.001	*p* > 0.05
	R^2^ 0.235	
6MWT	*p* < 0.001	*p* > 0.05
	R^2^ 0.294	
30STST	*p* < 0.001	*p* > 0.05
	R^2^ 0.317	

## Data Availability

The study protocol and de-identified individual participant data generated during this study are available from the investigators upon reasonable request with the publication. Requests should be directed to the corresponding author by email.
